# Tryptophan Metabolism Activates Aryl Hydrocarbon Receptor-Mediated Pathway To Promote HIV-1 Infection and Reactivation

**DOI:** 10.1128/mBio.02591-19

**Published:** 2019-12-17

**Authors:** Yan-Heng Zhou, Li Sun, Jun Chen, Wei-Wei Sun, Li Ma, Yang Han, Xia Jin, Qing-Xia Zhao, Taisheng Li, Hongzhou Lu, Xiu Qiu, Jian-Hua Wang

**Affiliations:** a The Joint Center for Infection and Immunity between Guangzhou Institute of Pediatrics, Guangzhou Women and Childrenˋs Medical Center, Guangzhou, China, and Institut Pasteur of Shanghai, Chinese Academy of Sciences (CAS), Shanghai, China; b CAS Key Laboratory of Molecular Virology and Immunology, Institut Pasteur of Shanghai, Chinese Academy of Sciences, Shanghai, China; c College of Life Sciences, Yan’an University, Yan’an, China; d Department of Infections and Immunity, Shanghai Public Health Clinical Center, Shanghai, China; e Department of Infectious Diseases, Peking Union Medical College Hospital, Chinese Academy of Medical Sciences, Beijing, China; f Department of Infection, Zhengzhou Sixth People’s Hospital, Zhengzhou, China; McMaster University

**Keywords:** HIV-1, aryl hydrocarbon receptor, tryptophan metabolite, transcription

## Abstract

Multiple cellular metabolic pathways are altered by HIV-1 infection, with an impact on immune activation, inflammation, and acquisition of non-AIDS comorbid diseases. The dysfunction of tryptophan (Trp) metabolism has been observed clinically in association with accelerated HIV-1 pathogenesis, but the underlying mechanism remains unknown. In this study, we demonstrated that the aryl hydrocarbon receptor (AHR), a ligand-activated transcription factor, is activated by Trp metabolites to promote HIV-1 infection and reactivation. AHR directly binds to the HIV-1 5′ long terminal repeat (5′-LTR) at the molecular level to activate viral transcription and infection, and AHR activation by Trp metabolites increases its nuclear translocation and association with the HIV 5′-LTR; moreover, the binding of AHR with HIV-1 Tat facilitates the recruitment of positive transcription factors to viral promoters. These findings not only elucidate a previously unappreciated mechanism through which cellular Trp metabolites affect HIV pathogenesis but also suggest that a downstream target AHR may be a potential target for modulating HIV-1 infection.

## Abstract (precis)

Cellular metabolic pathways that are altered by HIV-1 infection may accelerate disease progression. Dysfunction in tryptophan (Trp) metabolism has been observed clinically in association with accelerated HIV-1 pathogenesis, but the mechanism responsible was not known. This study demonstrates that Trp metabolites augment the activation of aryl hydrocarbon receptor (AHR), a ligand-activated transcription factor, to promote HIV-1 infection and transcription. These findings not only elucidate a previously unappreciated mechanism through which cellular Trp metabolites affect HIV pathogenesis but also suggest that a downstream target AHR may be a potential target for modulating HIV-1 infection.

## INTRODUCTION

The increased manifestation of metabolic comorbidities, such as obesity, type 2 diabetes, cardiovascular complications, neurodegeneration, and cancers, has emerged as a critical issue for clinical care of individuals with long-term HIV-1 infection (1–6). Host genetic factors, immunological status, antiretroviral drug treatments, aging, and behavior factors (such as smoking and diet) have been suggested to be risk factors for these non-AIDS comorbid diseases (7, 8). The persistent immune activation and inflammation caused by HIV-1 infection, and the resulting alteration of multiple cellular metabolic pathways of glucose, lipids, amino acids, and nucleotides, have become more extensively accepted as key contributing factors for the development of metabolic diseases (3, 4, 8, 9). On the one hand, immune dysfunction induced by HIV-1 infection causes irreversible damage of metabolic function, permanent organ system dysfunction, and microbial translocation at the intestinal mucosa (3, 10); on the other hand, the accumulating metabolites derived from aberrant metabolic pathways can flame further immune activation and inflammation (5, 11).

Recently, several studies have reported that viruses can manipulate cellular metabolisms, including glycolysis, fatty acid synthesis, and glutaminolysis, to arrange an optimized intracellular environment for productive viral infection and survival of infected cells (3, 4, 12, 13). Similarly, HIV-1 has been shown to promote fatty acid and cholesterol biosynthesis to support viral replication (14), and Nef protein is central to the increased cholesterol synthesis and transportation of cholesterol to lipid rafts which facilitates virus assembly (15). The dysfunctional cellular metabolisms are associated with faster CD4^+^ T cell depletion, rapid viral rebound, greater frequency of coinfection, and accelerated disease progression (3, 5). Thus, modulation of cellular metabolic pathways appears to be a reasonable approach to control HIV-1 disease progression. Therefore, in-depth understanding of HIV-associated metabolic modification may help to identify novel biomarkers and therapeutic targets.

l-Tryptophan (Trp) is an essential amino acid obtained exclusively from dietary intake in humans. Most free Trp can be metabolized into kynurenine (Kyn) via indoleamine 2,3-dioxygenase (IDO) (16, 17). A small fraction of the Trp that remains in the skin can also be converted into 6-formylindolo(3,2-b) carbazole (FICZ) by UV irradiation (18). Metabolism activities of Trp and several of its metabolites have key roles in diverse physiological processes and in a wide range of diseases, including central nervous system disorders, infectious diseases, autoimmune diseases, and cancer. For instance, Trp metabolites, including Kyn, could enhance cancer invasion and metastasis (19, 20); in various cancers such as colon cancer, lung cancer, breast cancer, melanoma, and brain tumors, the highly expressed IDO favors tumor growth and survival (17, 21, 22); in atherosclerosis and cardiovascular diseases, the enhanced IDO activity is positively correlated with production of essential risk factors of low-density lipoprotein and triglycerides but is negatively correlated with high-density lipoprotein, a protective factor (23, 24); and Trp metabolite quinolinic acid is neuroactive and its accumulation triggers disease progression in neurodegenerative diseases of Huntington disease, Parkinson disease, Alzheimer’s disease, and amyotrophic lateral sclerosis (17). Further, Kyn and its metabolite 3-hydroxybutyrateanthranilic acid can induce immunosuppression and ameliorate disease progression of autoimmune encephalomyelitis, autoimmune diabetes, and multiple sclerosis (25, 26). Thus, targeting Trp metabolism has led to the development of drugs for treatment of various human diseases (16, 17, 27).

In the context of HIV infection, clinical studies have revealed a correlation between the Trp-Kyn catabolism pathway and AIDS pathogenesis. Excessive IDO activity (measured by the Kyn/Trp ratio) in HIV-1-infected patients leads to tryptophan depletion and kynurenine accumulation (28–30). Higher ratios of Kyn/Trp have been observed to be positively associated with higher plasma virus loads, lower CD4^+^ T-cell counts, heightened chronic immune activation, increased immune suppression, enhanced microbial translocation, and a higher incidence of pathogen coinfection and comorbidities (27, 31–33). Moreover, our previous study demonstrated a positive correlation of the Kyn/Trp ratio with both viremia levels in treatment-naive patients and the size of the HIV-1 reservoir in patients subjected to combination antiretroviral treatment (cART) (34). cART can reduce the Kyn/Trp ratio, but it cannot normalize it (28–30, 35–37). Hence, the underlying mechanism responsible for the fact that elevated Kyn levels are associated with faster HIV-1 disease progression appears to be complex (38, 39). The relationship between FICZ, a photoproduct of Trp metabolism, and HIV-1 disease progression has been less extensively studied. Of note, UV radiation has been observed to induce the activation of HIV-1 transcription and replication (40, 41), and UV exposure is considered to be a cofactor that accelerates the progression of AIDS through inducing immunosuppression (42, 43). The data imply that certain metabolic products may play a role in the activation of HIV infection.

Both Kyn and FICZ can bind to aryl hydrocarbon receptor (AHR), a ligand-activated transcription factor which is involved in the regulation of a metabolism pathway that senses environmental toxins and endogenous ligands (44–46). AHR signaling also regulates the development, differentiation, and proliferation of cells (47, 48). It has recently become known that AHR can be used by viruses to promote their infection. For instance, AHR activation suppresses type I interferon-mediated innate defense to promote the replication of vesicular stomatitis virus (VSV), influenza virus (FluV) (A/Puerto Rico/8/1934 H1N1 strain), Sendai virus (SeV), and encephalomyocarditis virus (EMCV), herpes simplex virus 1 (HSV-1) (49). In HCV infection, the AhR-CYP1A1 (cytochrome p450 1A1) pathway is upregulated, resulting in the accumulation of enlarged lipid droplets which enhance the virus assembly; and AHR inhibitors reduce the production of infectious virions (50). In HIV-1 infection, AHR activation stimulated by ligand of 2,3,7,8-tetrachlorodibenzo-p-dioxin (TCDD) or by TCDD chemical homologue 3-methylcholanthrene (3-MC) was previously shown to reactivate HIV-1 from latency (51–54), and induction of AHR and AHR-regulated CYP1A1 enzymes by TCDD was shown to be associated with enhanced activity of HIV RNA-dependent DNA polymerase (Pol) and increased expression of viral protein in human T cells (55). Also, AHR-regulated CYP1A1 activation, induced by polycyclic aromatic hydrocarbons (PAH), a major constituent of cigarette smoke, has been implicated in HIV pathogenesis and disease progression (56).

To investigate various metabolites and their associations with HIV reservoir, we examined the Trp/Kyn AHR pathway status in HIV-infected patients and demonstrated specific interactions between HIV proteins and metabolic products of the AHR-mediated cellular pathways that facilitate HIV-1 infection and reactivation. These findings elucidate biochemical mechanisms through which AHR pathway and Trp metabolism exert influence on HIV pathogenesis and suggest potential host targets for modulating HIV-1 infection.

## RESULTS

### The plasma level of Kyn in HIV-1-infected individuals is negatively associated with CD4 T-cell counts and positively associated with viral load.

**FIG 1:**
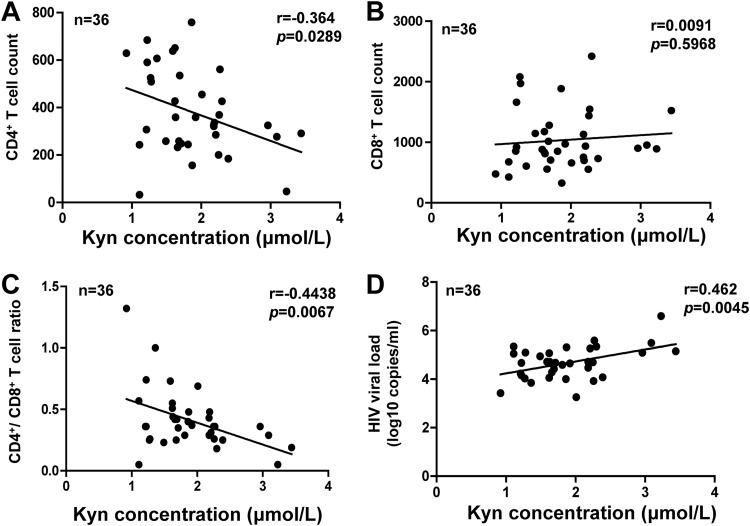
The plasma level of Kyn in HIV-1-infected individuals is negatively associated with CD4 T-cell counts and positively associated with viral load. The concentrations of Kyn CD4^+^ (A) and CD8^+^ (B) T cell counts and ratios (C) and viral load (D) in plasma from 36 treatment-naive HIV-1-infected participants were measured, and a Spearman correlation analysis was performed.

Abnormalities of tryptophan metabolism have been previously shown to be associated with disease progression in HIV-1-infected patients (28, 33). To verify those results, 36 treatment-naive HIV-1-infected participants were recruited, and the levels of tryptophan metabolite Kyn, viral load, and CD4^+^/CD8^+^ T-cell counts in plasma were examined (see Table S1 and S2 in the supplemental material). These patients were mostly male (34/36, 94.4%) and young (with a median age of 31 years; interquartile range [IQR], 26 to 37). The median Kyn concentration in patient plasma was 1.76 μΜ (IQR, 1.39 to 2.24), significantly higher than that in HIV-1-uninfected participants (1.43 μΜ; IQR, 1.15 to 1.76) (*P = *0.0043) (see Fig. S1 in the supplemental material). The correlations between Kyn levels and clinical parameters were analyzed with the Spearman rank correlation tests. The results showed that Kyn levels were negatively correlated with CD4^+^ T counts (*P = *0.0289) or with the CD4^+^/CD8^+^ T cell ratio (*P = *0.0067) (Fig. 1A to C) but were positively correlated with viremia levels (*P = *0.0045) (Fig. 1D).

10.1128/mBio.02591-19.1 FIG S1 Plasma Kyn concentration. The Kyn concentrations in plasma from 36 HIV-1-infected patients and 25 uninfected donors were quantified by using ultraperformance liquid chromatography-mass spectrometry. The plasma Kyn concentrations were analyzed in both groups. **, *P < *0.01 (considered the significant difference as determined by unpaired Student’s *t* test). Download FIG S1, TIF file, 0.01 MB. Copyright © 2019 Zhou et al. 2019 Zhou et al. This content is distributed under the terms of the Creative Commons Attribution 4.0 International license.

10.1128/mBio.02591-19.4 TABLE S1 Detailed information for treatment-naive participants. Download Table S1, TIF file, 0.02 MB. Copyright © 2019 Zhou et al. 2019 Zhou et al. This content is distributed under the terms of the Creative Commons Attribution 4.0 International license.

10.1128/mBio.02591-19.5 TABLE S2 Summarized information for treatment-naive patients. Download Table S2, TIF file, 0.01 MB. Copyright © 2019 Zhou et al. 2019 Zhou et al. This content is distributed under the terms of the Creative Commons Attribution 4.0 International license.

### Tryptophan metabolites reactivate HIV-1 in cells isolated from cART-treated patients.

**FIG 2:**
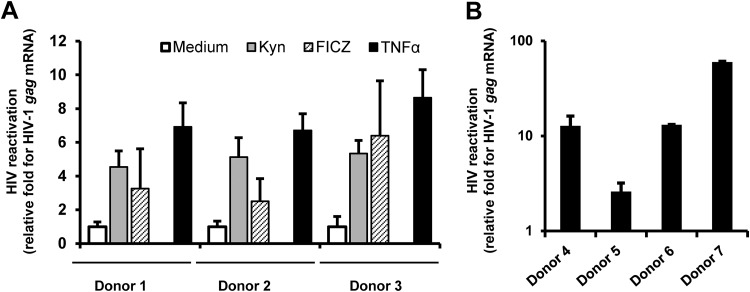
Tryptophan metabolites reactivate HIV-1 in cells isolated from cART-treated patients. (A) PBMCs isolated from cART-treated HIV-1 patients were treated with Kyn (200 μM) or FICZ (500 nM) for 4 days. TNF-α (1 μg/ml) stimulation was used as a positive control. (B) Purified resting CD4^+^ T cells from cART-treated HIV-1 patients were stimulated with FICZ (10 μM) for 4 days. Viral reactivation was measured by quantifying the production of intracellular *gag* mRNA for donors 1 to 3 (A) and donors 4 to 7 (B). The fold enrichment representing viral reactivation relative to medium treatment was calculated.

Because the Trp metabolite Kyn usually functions as an endogenous ligand of AHR (16, 19) and because the activation of AHR caused by Kyn might be an intermediate step leading to accelerated HIV-1 disease progression, we next investigated the effect of AHR ligands on HIV-1 replication. In addition to Kyn, a tryptophan photoproduct, FICZ, was examined. Peripheral blood mononuclear cells (PBMCs) were isolated from a panel of samples from cART-treated HIV-1 patients (Table S3) and were then treated with Kyn or FICZ for 4 days, at which time viral reactivation was measured by quantifying the production of intracellular *gag* mRNA. Tumor necrosis factor alpha (TNF-α) stimulation is known to reactivate HIV-1 and thus was used as a positive control, and unstimulated medium was used as a negative control. Results showed that both AHR ligands could reactivate HIV-1 in PMBCs. Compared with the unstimulated medium control, Kyn and FICZ caused 4.5-fold to 5.4-fold enhancement and 2.5-fold to 6.4-fold enhancement of the levels of *gag* mRNA production, respectively (Fig. 2A). To confirm that the data represented results of HIV reactivation, resting CD4^+^ T cells from cART-treated HIV-1 patients were purified and stimulated with a higher concentration of FICZ for 4 days and were then measured for *gag* mRNA production. Again, 6.8-fold to 70-fold enhancement was observed (Fig. 2B).

10.1128/mBio.02591-19.6 TABLE S3 Detailed information for cART-treated HIV-1 patients. Download Table S3, TIF file, 0.05 MB. Copyright © 2019 Zhou et al. 2019 Zhou et al. This content is distributed under the terms of the Creative Commons Attribution 4.0 International license.

To investigate whether the treatments performed with the compounds could induce the nonspecific activation of CD4^+^ T cells, which might also represent a susceptible target for new infection (57), the resting CD4^+^ T cells isolated from healthy donors were treated with higher concentrations of FICZ or Kyn for 4 days, and the levels of nonspecific activation of cells were evaluated by detecting surface CD69 expression. Results showed that the stimulation performed with AHR ligand FICZ or Kyn did not induce nonspecific activation of CD4^+^ T cell (Fig. S2).

10.1128/mBio.02591-19.2 FIG S2 Nonspecific activation of primary CD4^+^ T cells. PBMCs (2 × 10^5^) from healthy donors were treated with Kyn (200 μM) or FICZ (10 μM) for 4 days, and PHA-P (5 μg/ml) was used as the control. CD4^+^ T cells were gated, and CD69 expression on cell surface was detected with flow cytometry. Results from three independent donors are shown. Download FIG S2, TIF file, 0.1 MB. Copyright © 2019 Zhou et al. 2019 Zhou et al. This content is distributed under the terms of the Creative Commons Attribution 4.0 International license.

Taken together, these results prove that stimulation with AHR ligands represents a specific pathway that could reactivate HIV-1 from cART-suppressed patients.

### AHR assists HIV-1 infection.

**FIG 3:**
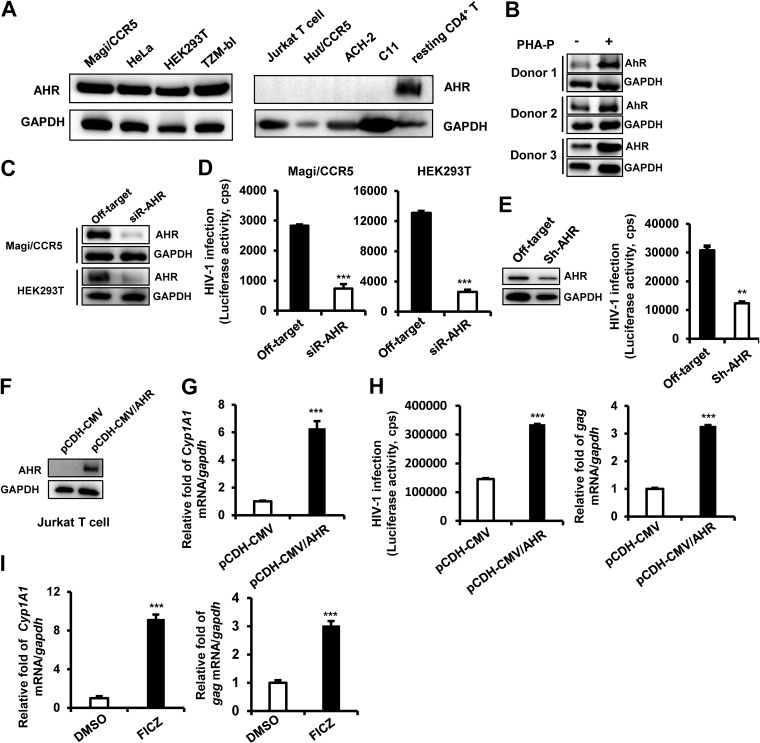
AHR promotes HIV-1 infection. (A) Endogenous expression of AHR in multiple transformed cells was detected by Western blotting. (B) AHR expression in primary CD4^+^ T cells. Primary resting CD4^+^ T cells (1 × 10^6^) were stimulated with or without 5 μg/ml of PHA-P for 72 h, and then endogenous expression of AHR was detected by Western blotting. (C and D) AHR knockdown impaired HIV-1 infection. Magi/CCR5 and HEK293T cells were transfected with AHR-specific siRNAs or off-target controls for 48 h, and then Magi/CCR5 cells were infected with HIV-luc/JRFL (2 ng p24*^gag^*) and HEK293T cells with HIV-luc/VSV-G (1 ng p24*^gag^*) for an additional 24 h. AHR expression was detected by Western blotting (C), and viral infections were quantified by detection of luciferase activity (D). (E) AHR knockdown inhibited HIV-1 infection of primary CD4^+^ T cells. PHA-P-activated primary CD4^+^ T cells (3 × 10^6^) were infected with lentivirus-containing AHR-specific shRNA or the off-target control for 48 h, and then cells were further infected with HIV-luc/NL4-3 (10 ng p24*^gag^*) for an additional 96 h. AHR expression and HIV infection were detected as described above. (F to H) AHR overexpression promoted HIV infection. Jurkat T cells were infected with lentivirus-containing AHR-expressing plasmid (pCDH-CMV/AHR) or vectors control. Puromycin was presented for selection. AHR expression was identified by Western blotting (F), the products of *CYP1A1* mRNA were quantified by (RT-)PCR (G), and cells were subsequently infected with HIV-luc/VSV-G (1 ng p24*^gag^*) for an additional 24 h, and viral infection was detected by measuring luciferase activity (H, left panel) and by quantitative (RT-)PCR analysis of *gag* mRNA transcripts (H, right panel). (I) AHR activation enhanced HIV-1 infection. PHA-P-activated primary CD4^+^ T cells (3 × 10^6^) were infected with HIV-luc/NL4-3 (10 ng p24*^gag^*) for 6 h, and then the cells were washed and further incubated in the presence of FICZ (100 nM) or dimethyl sulfoxide (DMSO) (0.1%) for an additional 96 h. The transcript of *CYP1A1* mRNA was quantified, and HIV-1 infection was measured by quantifying the transcript of *gag* mRNA. Data are presented as means ± standard deviations (SD). Data shown are from one experiment representative of results from three independent experiments.**, *P < *0.01; ***, *P < *0.001 (considered significant differences in an unpaired *t* test). cps, counts per second.

Having demonstrated a role of AHR ligands in reactivating HIV-1, we went on to investigate mechanistically how AHR modulates HIV-1 infection. The endogenous expression of AHR in multiple cell types was screened, and high expression levels were found in human embryonic kidney cells (HEK293T) T cells, HeLa cells, and HeLa-derived Magi/CCR5 and TZM-bl cells (Fig. 3A, left panel); no expression was found in transformed CD4^+^ T cells, including Jurkat T cells, Hut/CCR5 cells, and ACH2 and C11 clones (derived from Jurkat T cells) latently infected with HIV-1 (Fig. 3A, right panel). Notably, constitutive expression of AHR in primary resting CD4^+^ T cells was observed (Fig. 3A, right panel, and Fig. 3B), and phytohemagglutinin-P (PHA-P) stimulation elevated AHR expression in primary CD4^+^ T cells (Fig. 3B).

To investigate the role of AHR in HIV-1 infection, the endogenously expressed AHR in HEK293 T cells, Magi/CCR5 (Fig. 3C), and PHA-P-activated primary CD4^+^ T cells (Fig. 3E, left panel) was knocked down by using specific small interfering RNAs (siRNAs) or lentiviruses containing AHR-specific shRNAs, and then cells were infected with pseudotyped HIV-1. AHR knockdown significantly impaired HIV-luc/JRFL infection of Magi/CCR5 cells, HIV-luc/VSV-G infection of HEK293T cells (unpaired *t* test, *P < *0.001) (Fig. 3D), and HIV-luc/NL4-3 infection of primary CD4^+^ T cells (unpaired *t* test, *P < *0.01) (Fig. 3E, right panel).

In contrast, when AHR was stably overexpressed in Jurkat T cells by infection with lentivirus-containing AHR-expressing vector pCDH-CMV/AHR (Fig. 3F), AHR-mediated signaling could be detected by the enhanced downstream *CYP1A1* gene expression (Fig. 3G), and significantly increased HIV-luc/VSV-G infection was detected by increased luciferase activity and *gag* mRNA production (unpaired *t* test, *P < *0.001) (Fig. 3H). Knockdown of AHR mediated by siRNA in HEK293T cells or AHR overexpression mediated by lentivirus vector pCDH-CMV/AHR in Jurkat cells did not alter cell proliferation (Fig. S3A and B), which was assessed by using a 3-(4,5-dimethyl-2-thiazolyl)-2,5-diphenyl-2-H-tetrazolium bromide (MTT) colorimetric method as described previously (58).

10.1128/mBio.02591-19.3 FIG S3 Cell proliferation assay. A total of 2 × 10^4^ HEK293T cells transfected with AHR-specific siRNA or off-target controls (A) or 3 × 10^4^ cells Jurkat T cells transduced with lentivirus-containing AHR-expressing plasmid (pCDH-CMV/AHR) or vectors (B) were seeded in 96-well plates and incubated for the indicated times. Cell proliferation was assessed by using the MTT colorimetric method. Data are presented as means ± standard deviations (SD). Download FIG S3, TIF file, 0.02 MB. Copyright © 2019 Zhou et al. 2019 Zhou et al. This content is distributed under the terms of the Creative Commons Attribution 4.0 International license.

To further examine whether AHR activation is sufficient to promote HIV-1 infection, PHA-P-activated primary CD4^+^ T cells were first infected with HIV-luc/NL4-3 for 6 h and then further treated with FICZ for an additional 4 days. FICZ treatment activated AHR-mediated signaling, as shown by the significantly enhanced level of *CYP1A1* gene expression (Fig. 3I, left panel), and significantly increased the level of HIV-luc/NL4-3 infection (unpaired *t* test, *P < *0.001) (Fig. 3I, right panel). Taken together, these data demonstrate that AHR is a positive regulator of HIV-1 infection.

### AHR binds to HIV-1 5ˊ-LTR and promotes viral transcription.

**FIG 4:**
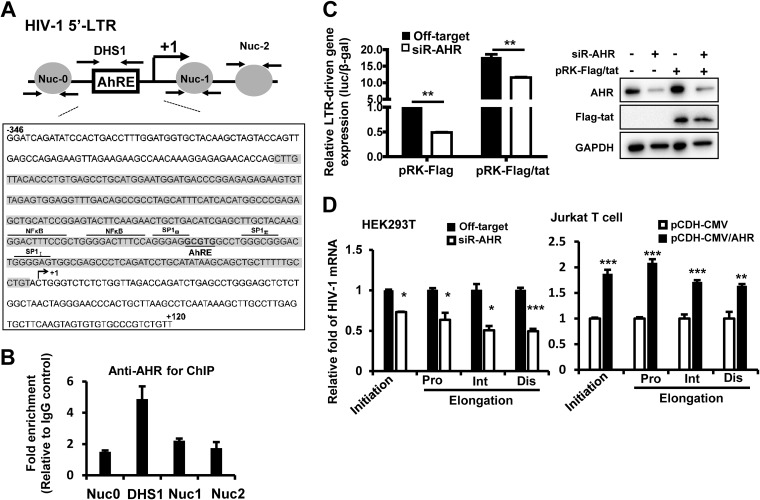
AHR binds to HIV-1 5ˊ-LTR and promotes viral transcription. (A) Schematic graph for HIV-1 5ˊ-LTR, showing the locations of nucleosomes (Nuc0, Nuc1, and Nuc2), DHS1, and AhRE. The fragment of the 5ˊ-LTR of HIV-1_NL4-3_ is presented in a zoomed-in image, showing the binding sites for NF-κB, SP, and AHR. The whole sequence for DHS1 was highlighted in gray shading. (B) AHR binding to the HIV-1 5ˊ-LTR analyzed by ChIP-PCR assay. HEK293T cells were infected with HIV-luc/VSV-G for 24 h, and then the cells were subjected to a cross-linked ChIP assay with antibodies against AHR or an IgG control, and the different fragments of 5ˊ-LTR were amplified by (RT-)PCR. (C) AHR enhanced HIV-1 5ˊ-LTR-driven gene expression. HEK293T cells were transfected with AHR-specific siRNA or an off-target control for 48 h, and then cells were further transfected with the plasmid containing a luciferase reporter driven by the full-length LTR promoter derived from HIV-1_NL4-3_ for 24 h. Some samples were cotransfected with HIV-1 *tat*-expressing plasmid pRK-Flag/tat or empty vector control. 5ˊ-LTR-driven gene expression was measured by quantifying luciferase activity normalized with β-galactosidase activity (left panel). AHR knockdown and Tat expression were detected by Western blotting (right panel). (D) AHR promoted both the initiation and elongation of HIV-1 transcription. HEK293T cells with or without AHR knockdown with siRNAs were infected with HIV-luc/VSV-G for 24 h (left panel), Jurkat T cell with or without AHR overexpression were infected with HIV-luc/JRFL for 24 h (right panel), and then both types of cells were harvested for total cellular RNA isolation, and HIV-1 transcript data, including transcription initiation (Ini) and proximal (Pro), intermediate (Int), and distal (Dis) elongation, were quantified with q(RT-)PCR with specific primers. Data are presented as means ± SD. Results are representative of three independent experiments. *, *P < *0.05; **, *P < *0.01; ***, *P* < 0.001 (considered significant differences in an unpaired *t* test).

We then investigated at the molecular level how AHR facilitated HIV replication. In its inactive form, AHR is retained in cytoplasm as a complex in association with chaperone protein HSP90, cochaperone protein p23, and an AHR-interacting protein (AIP) (47). Upon activation with ligands, AHR translocates to the nucleus and forms a heterodimer with an AHR nuclear translocator (ARNT) to regulate gene transcription (59). The AHR response element (AhRE) contains a “5**ˊ**-GCGTG-3**ˊ**” motif and locates at the promoter regions of genes, and it may be targeted by AHR, providing a scaffold for AHR/ARNT binding (60). Thus, we first examined whether the HIV-1 5ˊ long terminal repeat (5ˊ-LTR) contains an AhRE. By sequence analysis, we found the 5**ˊ**-GCGTG-3**ˊ** motif of AhRE embedded among binding sites of Sp transcription factors (Fig. 4A). SP binding elements locate at the 3ˊ terminus of the DNase hypersensitive site 1 (DHS1) region and representre the core region of 5ˊ-LTR. We performed a chromatin immunoprecipitation (ChIP)-PCR assay to show binding of AHR with HIV-1 5ˊ-LTR by using HIV-1-luc/VSV-G-infected HEK293T cells. As expected, AHR bound specifically to the DHS1 region, because immunoprecipitation with anti-AHR antibody mainly enriched the DHS1 region but not other LTR regions such as Nuc0, Nuc1, or Nuc2 (Fig. 4B).

To assess whether such binding has functional significance, we next investigated whether AHR could modulate HIV-1 5ˊ-LTR-driven transcription. HEK293T cells with or without AHR knockdown were transfected with a plasmid containing a luciferase reporter gene driven by the full-length LTR promoter derived from HIV-1_NL4-3._ Results showed that AHR knockdown significantly impaired HIV-1 5ˊ-LTR-driven basal transcription (unpaired *t* test, *P < *0.01) (Fig. 4C). Because HIV-1 Tat protein binds to a *trans*-activation response (TAR) element to drive transcription elongation, we further investigated whether AHR modulation of HIV gene transcript was mediated through the Tat-TAR axis by transfecting HEK293T cells with or without AHR knockdown using an HIV-1-*tat*-expressing plasmid (pRK-Flag/tat; cloned from HIV-1_NL4-3_). Results showed that AHR knockdown significantly impaired Tat-driven LTR activity (unpaired *t* test, *P < *0.01) (Fig. 4C).

Because the initiation and elongation of HIV-1 LTR-driven transcription can be assessed by quantifying transcripts of various lengths of HIV mRNA with PCR using specific primers (61–63), we also examined the relative lengths of the PCR products and found that AHR knockdown in HEK293T cells significantly shortened the length of each PCR product, suggesting a blockade of both the initiation and the elongation of HIV-1 5ˊ-LTR-driven transcription (unpaired *t* test, *P < *0.05 and *P < *0.001, respectively) (Fig. 4D, left panel). The reverse was also true in that AHR overexpression in Jurkat T cells markedly increased both the initiation and the elongation of HIV-1 transcription (unpaired *t* test, *P < *0.01 and *P < *0.001, respectively) (Fig. 4D, right panel). Taken together, these data demonstrate that AHR binds to the HIV-1 5ˊ-LTR to promote viral transcription.

### HIV-1 Tat uses AHR to assist the recruitment of P-TEFb complex to phosphorylate RNA Pol II.

Ligand-activated AHR binds the AhRE located at the promoter regions of AHR-targeted genes, initiating chromatin remodeling and recruitment of positive transcription factors to form the preinitiation complex and starting the elongation of transcription (47). Both the positive transcription elongation factor (P-TEFb) and phosphorylated RNA polymerase II (Pol II) have been shown to be recruited by ligand-activated AHR, and the association of AHR with P-TEFb is mediated through the C terminus of cyclin T1 (CycT1), a component of the P-TEFb complex (64).

**FIG 5:**
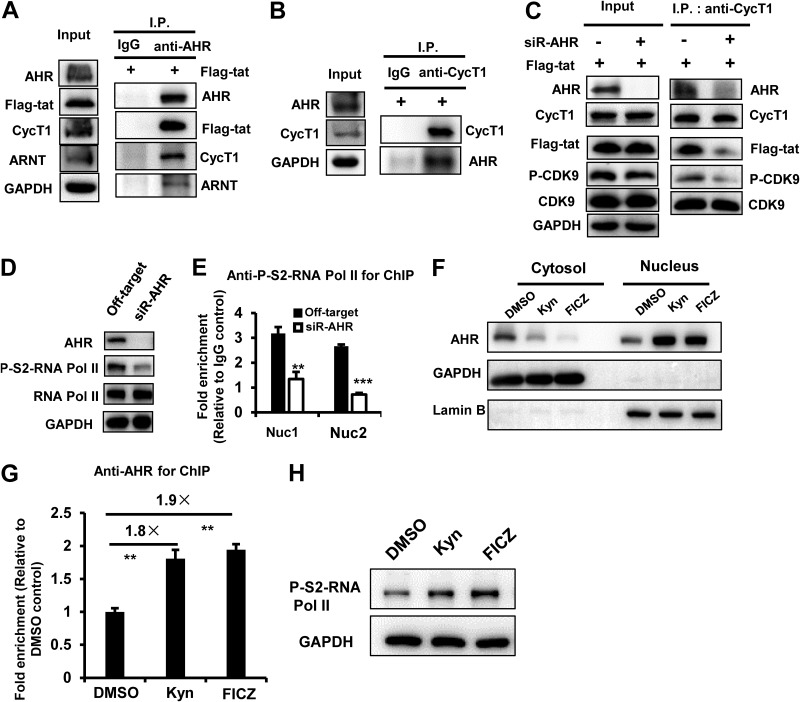
HIV-1 Tat exhibits AHR-dependent recruitment of P-TEFb complex for subsequent phosphorylation of RNA Pol II. (A and B) AHR forms complexes with HIV-1 Tat, cyclin T1, and ARNT. (A) HEK293T cells were transfected with pRK-Flag/tat plasmid for 24 h, cell lysates were subjected to immunoprecipitation (I.P.) by using anti-AHR-specific antibody or an IgG control, and immunoblotting was performed by using the indicated antibodies. (B) The anti-CycT1-specific antibody was used for immunoprecipitation. (C) AHR knockdown impaired Tat-mediated recruitment of P-TEFb. HEK293T cells with or without AHR knockdown with siRNAs were transfected with pRK-Flag/tat plasmid for 24 h. Immunoprecipitation was performed with anti-CycT1-specific antibody, and immunoblotting was performed by using the indicated antibodies. (D and E) AHR knockdown decreased the phosphorylation of RNA Pol II and the binding of phosphorylated RNA Pol II to the HIV-1 5ˊ-LTR. HEK293T cells with or without AHR knockdown were infected with HIV-luc/VSV-G for 24 h, the phosphorylation of RNA Pol II at the serine 2 of CTD domain was measured with Western blotting using specific antibody (D), and the association of phosphorylated RNA Pol II with HIV-1 5ˊ-LTR was measured by ChIP-PCR assay using nuc1- and nuc2-specific primers (E). (F to H) Ligand treatment enhanced AHR nuclear translocation, association with the DHS1 region of 5ˊ-LTR, and phosphorylation of RNA Pol II. HEK293T cells were infected with HIV-luc/VSV-G for 24 h and then treated with Kyn (200 μM), FICZ (100 nM), or DMSO (0.1%) for 30 min. Cells were collected, and the cytosol and nucleus components were fractionated and immunoblotted with the indicated specific antibodies (F), ChIP-PCR assay was conducted using DHS1-specific primers to measure the association of AHR with HIV-1 5ˋ-LTR (G), and the whole-cell lysates were used to detect the phosphorylation of RNA Pol II (H). Results are representative of three independent experiments. **, *P < *0.01; ***, *P* < 0.001 (significant difference in an unpaired *t* test). CycT1, cyclin T1.

HIV-1 Tat protein has been known to recruit P-TEFb to stimulate viral transcription (65). We therefore examined whether Tat could use AHR-mediated recruitment of the P-TEFb complex. The immunoprecipitation assay demonstrated that AHR could form a complex with Tat, cyclin T1, and the cofactor of ARNT (Fig. 5A and B). To conclusively determine the AHR dependency of Tat-mediated recruitment of P-TEFb, AHR was knocked down in HEK293T cells and Tat-expressing plasmid was transfected into these cells. The immunoprecipitation assay showed that AHR knockdown markedly reduced the association of Tat with cyclin T1 (Fig. 5C). Notably, AHR knockdown did not affect the expression and phosphorylation of CDK9 but specifically reduced the association between phosphorylated CDK9 and cyclin T1 (Fig. 5C).

Because the recruited P-TEFb complex phosphorylates serine-2 (S2) at the C-terminal domain (CTD) of RNA Pol II for subsequent transcriptional elongation (65), we also examined whether AHR affects RNA Pol II and found that AHR knockdown reduced the phosphorylation of RNA Pol II (Fig. 5D) and significantly diminished the binding of phosphorylated RNA Pol II with HIV-1 5ˊ-LTR in HIV-luc/VSV-G-infected HEK293T cells (unpaired *t* test, *P < *0.01 and *P < *0.001, respectively) (Fig. 5E).

To further investigate the upstream events leading to the mediation of RNA Pol II phosphorylation by AHR activation, we next examined whether ligands of AHR might trigger the phosphorylation of RNA Pol II. We first assessed the translocation of the endogenous AHR upon stimulation using HEK293T cells infected with HIV-luc/VSV-G by treating them with Kyn or FICZ for 30 min and found that the ligand treatments increased both the translocation of AHR from the cytoplasm to the nucleus (Fig. 5F) and the association between AHR and the DHS1 region of 5ˋ-LTR (unpaired *t* test, *P < *0.01) (Fig. 5G); as a consequence, there was enhanced phosphorylation of RNA Pol II (Fig. 5H).

Taken together, the data presented above demonstrate that AHR-dependent recruitment of P-TEFb complex and the subsequent phosphorylation of RNA Pol II are linked to HIV-1 Tat, which, upon binding to TAR, promotes HIV-1 replication and accelerates HIV pathogenesis.

## DISCUSSION

Alterations of metabolic pathways within different cell types during HIV-1 infection have been associated with regulations of chronic immune activation, inflammation, disease progression, the acquisition of coinfections, and the occurrence of comorbidities (3–5). For instance, increased glucose metabolism in HIV infection was demonstrated 2 decades ago by a number of studies. One showed induced expression of glucose transporter-3 (Glut-3) and increased glucose uptake in the HIV-1-infected H9 human T-cell line *in vitro* (66). Another reported that *in vivo*, circulating CD4^+^ T cells from HIV-positive individuals exhibit a glycolytic phenotype featuring a substantially increased level of cell surface expression of Glut-1 and that they have abnormally high levels of hexokinase activity and lactate production. Furthermore, the CD4^+^ Glut-1^+^ T cells take up more glucose and show higher glycolytic activity, and the frequency of this type of cell is positively associated with markers of T cell activation and is inversely correlated with the percentages and absolute numbers of CD4^+^ T cells, irrespective of whether or not the patients were administered with antiretroviral treatment (67).

Alterations of metabolic pathways have been attributed to specific HIV proteins. For instance, overexpression of HIV-1 Vpr was shown to modulate multiple metabolic pathways in macrophages in proteomic studies using stable-isotope labeling by amino acids in cell culture (SILAC) coupled with a mass spectrometry approach (68). Dysregulation of mitochondrial glutamate metabolism, featuring increased glutamate production and release upon activation of glycolysis and the tricarboxylic acid cycle, might contribute to neurodegeneration via excitotoxic mechanisms in the context of neurological complications of AIDS (NeuroAIDS) (6). In previous studies using mouse models with transgenic overexpression or systemic infusion, Vpr was shown to block the expression of peroxisome proliferator–activated receptor γ but to stimulate glucocorticoid receptor-mediated signaling, resulting in dysfunction of fat metabolism that manifested as accelerated whole-body lipolysis, hyperglycemia, hypertriglyceridemia, and hepatosteatosis (69).

**FIG 6:**
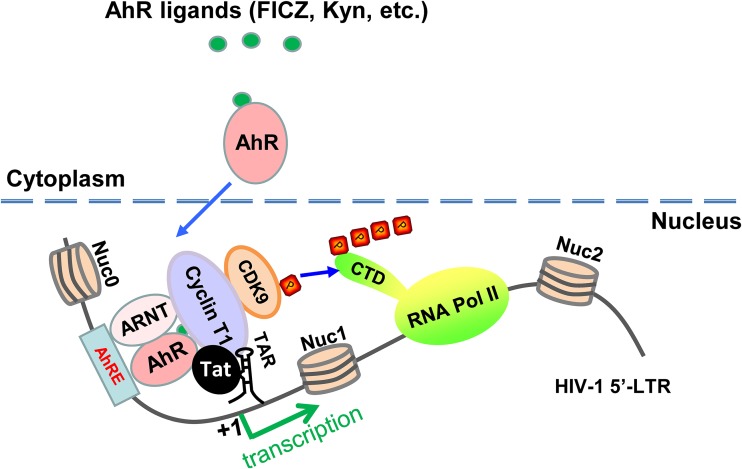
Schematic illustration of AHR activation modulating HIV-1 transcription. AHR activation stimulated with Trp metabolites (FICZ and Kyn) increases AHR nuclear translocation; accumulation of heterodimers of AHR/ARNT at the nucleus facilitates Tat-mediated recruitment of P-TEFb to HIV-1 5ˊ-LTR for enhancing the phosphorylation of RNA Pol II and its association with the 5ˊ-LTR promoter. CTD, C-terminal domain of RNA Pol II; Nuc: nucleosome; TAR, transactivation response element.

In this study, we enrich the existing literature by demonstrating that Trp metabolites, especially Kyn, increase HIV-1 viremia and decrease CD4^+^ T cell counts through activating AHR and facilitating Tat-TAR-initiated HIV-1 gene transcription (Fig. 6). These results reveal a new molecular mechanism of metabolic disorders in patients with HIV-1 infection and offer a molecular explanation for the often observed association between cellular Trp metabolism and HIV pathogenesis. In fact, the other metabolic components of Trp/Kyn pathway have been reported to play a role in HIV-1 disease progression, particularly in HIV-1-associated neurodegeneration. The significantly increased production of quinolinic acid, a neurotoxic metabolic intermediate of the Trp/Kyn pathway, has been observed in the brain and cerebrospinal fluid of AIDS patients, especially in those with AIDS dementia complex (ADC) (70), and the level of quinolinic acid is positively correlated with ADC occurrence and severity, and cART could decease quinolinic acid production and lead to neurological improvement in clinical settings (71, 72).

This and previous studies have indicated that targeting the metabolic pathway of immune cells may represent a new therapeutic approach to treat HIV diseases. For example, because glucose metabolism in immune cells is coordinated in part by phosphatidylinositol 3-kinase/Akt (PI3K/Akt), which posttranslationally regulates Glut1 trafficking to the cell membrane (73), experimental use of PI3K inhibitor LY294002 suppressed Glut1 surface expression and glucose uptake, leading to subsequent abrogation of HIV-1 infection of CD4^+^ T cells (74). Given the pivotal role of Trp metabolism in regulating diverse physiological processes and a wide range of infectious and immune diseases, targeting Trp metabolism has resulted in pharmacological intervention in the treatment of several diseases (16, 17, 27). In this study, we demonstrated that Trp metabolites Kyn and FICZ, both of which are ligands of AHR, can reactivate HIV-1 from reservoir cells isolated from antiretroviral-therapy-treated patients with sustained viral load suppression and undetectable viremia, suggesting the potential use of both compounds as the “shock” agents in the “shock and kill” strategy for HIV-1 eradication. In agreement with our data, a high-affinity AHR ligand, TCDD, has also been previously shown to play a role in reactivating HIV-1 (51–54).

FICZ is a photoproduct of Trp seen upon UV irradiation (18), and it has received much attention because of its high-affinity binding to AHR and consequential regulation of the expression of many genes involved in viral infection and cancers (49, 75). UV radiation was proposed 2 decades ago to be a cofactor which accelerates the progression of AIDS by inducing immunosuppression. It gradually become known that UV radiation increases TNF-α production and leads to the accumulation of *cis*-urocanic acid, a potent immunosuppressant (42), and that UV exposure activates HIV-1 transcription and replication in cultured cells, transgenic mice, and human skin tissues of those HIV-infected patients with UV-treatable skin disorders (41, 76–78). In this study, we uncovered a new molecular mechanism responsible for UV-mediated pathogenesis: that photoproduct FICZ promotes HIV-1 reactivation via regulating AHR activation.

The discovery of AHR playing a role in HIV-1 replication has potential clinical significance. AHR is a ligand-dependent transcriptional factor widely expressed among immune, epithelial, endothelial, and stromal cells in barrier tissues. In inactive status, AHR remains in cytoplasm as a complex in association with a chaperone protein (HSP90), a cochaperone protein (p23), and an AHR-interacting protein (AIP) (47). AHR activation results in its translocation to the nucleus to form a dimer with ARNT and thus in control of the expression of target genes with AhREs at the promoter region. In this study, we found that HIV-1 5ˊ-LTR harbored the AhREs, which provide the structural base for AHR binding. Targeting the promoter, AHR has previously been shown to recruit positive transcription factors P-TEFb and the phosphorylated RNA Pol II (64). We show here that HIV-1 Tat protein could associate with AHR and recruit positive transcriptional factors to promote infection. Given the participation and pivotal role of AHR-mediated gene transcription in a variety of physiological and pathological processes, targeting AHR provides therapeutic approaches for treating a range of autoimmune, neoplastic, and neurodegenerative diseases (75).

In summary, in this study, we demonstrated that normal cellular and molecular pathways mediated by AHR were used by HIV-1 to promote infection and viral reactivation and that Trp metabolites augmented the efficiency of this process. The finding that AHR is playing a role in the modulation of HIV-1 infection can explain the correlation between cellular Trp metabolism and HIV pathogenesis, and it also provides new host targets for the treatment of HIV-1 infection.

## MATERIALS AND METHODS

### Ethics statement and the treatments of human cells.

The usage of human samples and the related methods and experimental protocols have been approved by the Medical Ethics Review Committee of Institut Pasteur of Shanghai, Chinese Academy of Sciences, the Medical Ethics Review Committee of the Peking Union Medical College Hospital, and the Shanghai Public Health Clinical Center. All experiments were performed in accordance with relevant national guidelines and regulations.

Plasma samples were collected from 36 cART-naive HIV-1-infected participants from among the outpatients of the Shanghai Public Health Clinical Center, Shanghai, China. CD4^+^ and CD8^+^ T-cell counts were analyzed by flow cytometry (BD Biosciences). HIV-1 loads were quantified by PCR (Cobas Amplicor). Kynurenine concentrations were quantified by using ultraperformance liquid chromatography-mass spectrometry as previously described (79).

Peripheral blood mononuclear cells (PBMCs) from cART-treated HIV-1-infected patients were collected by Ficoll-Paque density gradient centrifugation (GE Healthcare Life Sciences). These patients had undergone cART comprised of administration of TDF (tenofovir disoproxil fumarate), 3TC (lamivudine), and EFV (efavirenz) for more than 2 years, with an undetectable HIV-1 load in the plasma. These patients are outpatients from the Peking Union Medical College Hospital, Beijing, China. In some samples, the resting CD4^+^ T cells were purified from PBMCs by using anti-CD4 antibody-coated magnetic beads (Miltenyi Biotec). Cells were treated with various concentrations of l-kynurenine (Kyn) (Sigma-Aldrich), 6-formylindolo(3,2-b) carbazole (FICZ) (Sigma-Aldrich), and TNF-α for 4 days, and then viral reactivation was measured by quantifying the production of intracellular *gag* mRNA and the enhancement relative to the level seen with a medium treatment control was calculated.

### Cells culture.

PBMCs from healthy donors were purchased from the Shanghai Blood Center (Shanghai, China). CD4^+^ T cells were purified from PBMCs by using anti-CD4 antibody-coated magnetic beads (Miltenyi Biotec, Germany). Resting CD4^+^ T cells were activated by treating them with 5 μg/ml phytohemagglutinin-P (PHA-P) (Sigma-Aldrich) for 3 days in the presence of 20 IU/ml recombinant interleukin-2 (IL-2; R&D Systems). CD4^+^ T-lymphocyte cell line Hut/CCR5 and Jurkat T cells were grown in RPMI 1640 medium with 10% fetal bovine serum (FBS) (Gibco) and with 100 U/ml penicillin and 100 μg/ml streptomycin (Invitrogen). Human embryonic kidney cells (HEK293T), HeLa cells, and HeLa-derived Magi/CCR5 and TZM-bl cells were cultured in Dulbecco’s modified Eagle medium (DMEM) (HyClone) containing 10% fetal bovine serum (FBS) (HyClone) and 100 U/ml penicillin and 100 μg/ml streptomycin. Cells were grown at 37°C under 5% CO_2_.

### Plasmids, siRNA, and shRNA.

The full-length human AHR gene encoding 848 amino acids was cloned into lentiviral vector pCDH-CMV. pGL3-LTR-luc (kindly provided by Li Wu, The Ohio State University, USA) was constructed by PCR amplification of the corresponding DNA fragment from pNL4-3 and subsequently cloned into pGL3-basic vector (Promega). The pRK-Flag/tat was kindly provided by De-Yin Guo (Wuhan University, China) (80). The specific siRNA targeting AHR (siR-AHR) was 5ˊ-GGG AAA GAU GGA UCA AUA C-3ˊ (Gene Pharma, Shanghai, China). Lipofectamine 2000 (Life Technologies) was used for siRNA and plasmid transfection according to the manufacturer′s protocol. The short hairpin RNA (shRNA) used for targeting AHR was 5ˊ-ATC CAC AGT CAG CCA TAA TAA-3ˊ, and the off-target shRNA control was 5ˊ-TTC TCC GAA CGT GTC ACG TAT-3ˊ. AHR shRNAs were subcloned into pLKO.1-puro shRNA expression vector. Calcium phosphate-mediated transfection of HEK293T cells was used to generate lentiviruses containing AHR shRNA or AHR-expressing plasmid (pCDH-CMV/AHR).

### HIV-1 stocks.

Calcium phosphate-mediated transfection of HEK293T cells was used to generate virus stocks. Pseudotyped single-cycle infectious HIV-luc/VSV-G, HIV-luc/NL4-3 (CXCR4 tropic), or HIV-luc/JRFL (CCR5 tropic) was obtained by cotransfection with luciferase reporter HIV-1 proviral plasmid pLAI-Δ-env-luc and the expression plasmid corresponding to vesicular stomatitis virus G (VSV-G) protein, HIV-1_NL4-3_ Env, or HIV-1_JR-FL_ Env, respectively. Harvested supernatants that contained viral particles were filtered and quantified by p24*^gag^* capture enzyme-linked immunosorbent assay (ELISA). The HIV-1 p24*^gag^* specific monoclonal antibodies were a kind gift from Yong-Tang Zheng (Kunming Institute of Zoology, Chinese Academy of Sciences, China). Viral infection was measured by detecting luciferase activity using a Luciferase assay system (Promega) or real-time PCR (RT-PCR) to detect viral *gag* mRNA expression.

### Real-time PCR (RT-PCR).

Total cellular mRNA was extracted using TRIzol reagent (Life Technologies) and then reverse transcribed into cDNA using ReverTra Ace quantitative PCR (qPCR) reverse transcription master mix with a gDNA remover kit (Toyobo). RT-PCR was performed using Thunderbird SYBR qPCR mix (Toyobo) and an ABI 7900HT real-time PCR system, with an initial denaturation step performed for 5 min at 95°C and amplification with 40 cycles of denaturation (95°C for 30 s) followed by annealing (60°C for 30 s). The data were analyzed by the use of SYBR green (Toyobo) and were semiquantified and normalized with GAPDH (glyceraldehyde-3-phosphate dehydrogenase) (61).The primers used for *gag* and GAPDH were as follows: for *gag*, forward primer 5ˊ-GTG TGG AAA ATC TCT AGC AGT GG-3ˊ and reverse primer 5ˊ-CGC TCT CGC ACC CAT CTC-3ˊ; for GAPDH, forward primer 5ˊ-ATC CCA TCA CCA TCT TCC AGG-3ˊ and reverse primer 5ˊ-CCT TCT CCA TGG TGG TGA AGA C-3ˊ. The initial (Ini) primers used to target base pairs (bp) 10 to 59 of the HIV-1 transcript were as follows: forward primer 5ˊ-GTT AGA CCA GAT CTG AGC CT-3ˊ and reverse primer 5ˊ-GTG GGT TCC CTA GTT AGC CA-3ˊ. The proximal (Pro) primers used to target bp 29 to 180 of the HIV-1 transcript were as follows: forward primer 5ˊ-TGG GAG CTC TCT GGC TAA CT-3ˊ and reverse primer 5ˊ-TGC TAG AGA TTT TCC ACA CTG A-3ˊ. The intermediate (Int) primers used to target bp 836 to 1015 of the HIV-1 transcript were as follows forward primer 5ˊ-GTA ATA CCC ATG TTT TCA GCA TTA TC-3ˊ and reverse primer 5ˊ-TCT GGC CTG GTG CAA TAG G-3ˊ. The distal (Dis) primers used to target bp 2341 to 2433 of the HIV-1 transcript were as follows: forward primer 5ˊ-GAG AAC TCA AGA TTT CTG GGA AG-3ˊ and reverse primer 5ˊ-AAA ATA TGC ATC GCC CAC AT-3ˊ.

### Immunoprecipitation and immunoblotting.

Cells were lysed in radio immunoprecipitation assay (RIPA) buffer {50 mM HEPES [4-(2-hydroxyethyl)-1-piperazineethanesulfonic acid] (pH 7.4), 150 mM NaCl (sodium chloride), 0.5 mM EGTA [ethylene glycol-bis (β-aminoethyl ether)-N,N,Nˊ,Nˊ-tetraacetic acid], 1% protease inhibitor cocktail (Sigma), 1 mM sodium orthovanadate, 1 mM NaF (sodium fluoride), 1% (vol/vol) Triton X-100, and 10% (vol/vol) glycerol} for 1 h on ice with brief vortex mixing performed every 10 min. After centrifugation for 10 min at 12,000 × g, the lysates were incubated with the indicated antibody at 4°C overnight. Protein G/A-labeled Dynabeads were added to each sample at 4°C for 2 h for immunoprecipitation. The immunoprecipitates were separated by SDS (sodium dodecyl sulfate)-PAGE (polyacrylamide gel electrophoresis) and analyzed by immunoblotting. Nuclear and cytoplasmic protein fractions were purified by using NE-PER nuclear and cytoplasmic extraction reagents (Thermo Scientific) according to the instructions of the manufacturer.

For immunoblotting, cells were lysed for 1 h at 4°C in ice-cold RIPA buffer. After centrifugation for 10 min at 12,000 × g, the supernatant was boiled in reducing SDS sample loading buffer and was analyzed by SDS-PAGE. Specific primary antibodies were used, followed by horseradish peroxidase-conjugated goat anti-mouse IgG or goat anti-rabbit IgG (Sigma) as the secondary antibody. A total of 5% of the lysates was used as the input.

### Chromatin immunoprecipitation (ChIP)-PCR.

ChIP experiments were performed according to a protocol provided with an EZ-ChIP chromatin immunoprecipitation kit (Millipore). Briefly, HEK293T or HEK293T cells with AHR knockdown were infected with HIV-luc/VSV-G for 24 h. In some tests, cells were further treated with Kyn or FICZ as indicated. Cells were cross-linked with 1% formaldehyde for 10 min at room temperature and quenched with 0.125 M glycine for 5 min. After lysis, nuclear extracts were separated and chromatin was sheared by the use of a sonicator (Bioruptor UCD-200; Diagenode) for 10 min (10 s on, 10 s off) on ice to obtain DNA fragments that were 200 to 1,000 bp in length. One percent of total sheared chromatin DNA was used as the input. Nuclear extracts were incubated with the indicated antibodies overnight at 4°C. Rabbit IgG was used as a negative control. Protein A/G-labeled magnet beads were added to each sample at 4°C for 4 h for immunoprecipitation. The immunoprecipitated DNA was analyzed through the use of real-time PCR (ABI Prism 7900 real-time PCR system) for 40 cycles with Thunderbird SYBR qPCR mix (Toyobo). The primers targeting the HIV-1 LTR Nuc0, DHS1, Nuc1, and Nuc2 regions have been described previously (81) and were as follows: for Nuc0, forward primer 5ˊ-TGG ATC TAC CAC ACA CAA GG-3ˊ and reverse primer 5ˊ-GTA CTA ACT TGA AGC ACC ATC C-3ˊ; for DHS1, forward primer 5ˊ-AAG TTT GAC AGC CTC CTA GC-3ˊ and reverse primer 5ˊ-CAC ACC TCC CTG GAA AGT C-3ˊ; for Nuc1, forward primer 5ˊ-TCT CTG GCT AAC TAG GGA ACC-3ˊ and reverse primer 5ˊ-CTA AAA GGG TCT GAG GGA TCT C-3ˊ; for Nuc2, forward primer 5ˊ-AGA GAT GGG TGC GAG AGC-3ˊ and reverse primer 5ˊ-ATT AAC TGC GAA TCG TTC TAG C-3ˊ.

### Antibodies.

The following antibodies were used for ChIP or immunoblotting: anti-AHR (83200 s; Cell Signaling Technology); anti-CycT1 (81464; Cell Signaling Technology); anti-CDK9 (2316; Cell Signaling Technology); anti-phospho-CDK9 (Thr186) (2549; Cell Signaling Technology); anti-RNA polymerase II CTD (C-terminal domain) repeat YSPTSPS (phospho S2) (ab5095; Abcam); anti-RNA polymerase II (05-952; Merck Millipore); anti-GAPDH (M20006; Abmart); anti-Flag (F1804; Sigma); anti-lamin B1 (66091-1-Ig; Proteintech).

### Flow cytometry to detect CD69 expression.

Peripheral blood mononuclear cells (PBMCs) from healthy donors were collected with Ficoll-Paque density gradient centrifugation (GE Healthcare Life Sciences). A total of 2 × 10^5^ PBMCs were treated with Kyn (200 μM) or FICZ (10 μM) for 4 days, and stimulation with PHA-P (5 μg/ml) was used as the control. CD4^+^ T cells were gated, and CD69 expression on cell surface was detected with flow cytometry. The specific antibodies phycoerythrin (PE)-anti-CD4 monoclonal antibody (RPA-T4; eBioscience) and fluorescein isothiocyanate (FITC)-anti-human CD69 antibody (FN50; eBioscience) were used. Cells were detected using a Fortessa flow cytometer (BD Pharmingen), and data were analyzed with FlowJo 7.6.1 software.

### Cell proliferation assay.

A total of 2 × 10^4^ HEK293T cells transfected with AHR-specific siRNA or off-target controls or 3 × 10^4^ Jurkat T cells transduced with lentivirus-containing AHR-expressing plasmid (pCDH-CMV/AHR) or vectors were seeded in a 96-well plate and incubated for the indicated times. Cell proliferation was assessed by using an MTT colorimetric method as described previously (58). In brief, 20 μl MTT [3-(4,5-dimethyl-2-thiazolyl)-2,5-diphenyl-2-H-tetrazolium bromide] (5 mg/ml) was added to cells for 4 h, and then 100 μl 50% dimethylformamide (DMF)–10% SDS was added overnight to confirm the complete dissolution of formazan. The absorbance values were read at 595 nm, and the reference value used was 630 nm. Cell proliferation was calculated, and the cell viability of samples transfected with off-target siRNA or vectors at the seeded time was normalized as 100%.

### Statistical analysis.

Statistical analysis was performed using the Wilcoxon signed-rank test. The Spearman rank correlation test was used to identify associations.
