# Supplementary figures and images for: Tryptophan Metabolism Activates Aryl Hydrocarbon Receptor-Mediated Pathway To Promote HIV-1 Infection and Reactivation
Source: mBio. 2019 Dec 17;10(6):e02591-19. doi: 10.1128/mBio.02591-19 (PMC6918076; doi:10.1128/mBio.02591-19)

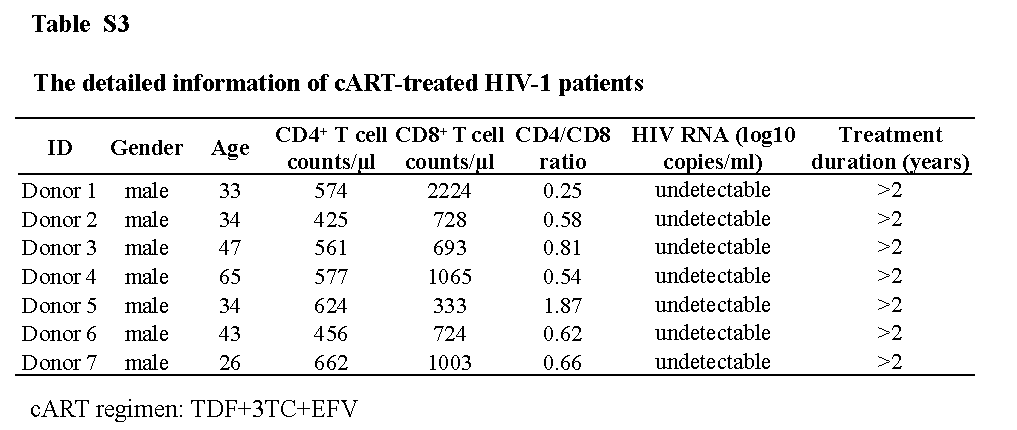

Supplement: TABLE S3 [file mBio.02591-19-st003.tif]

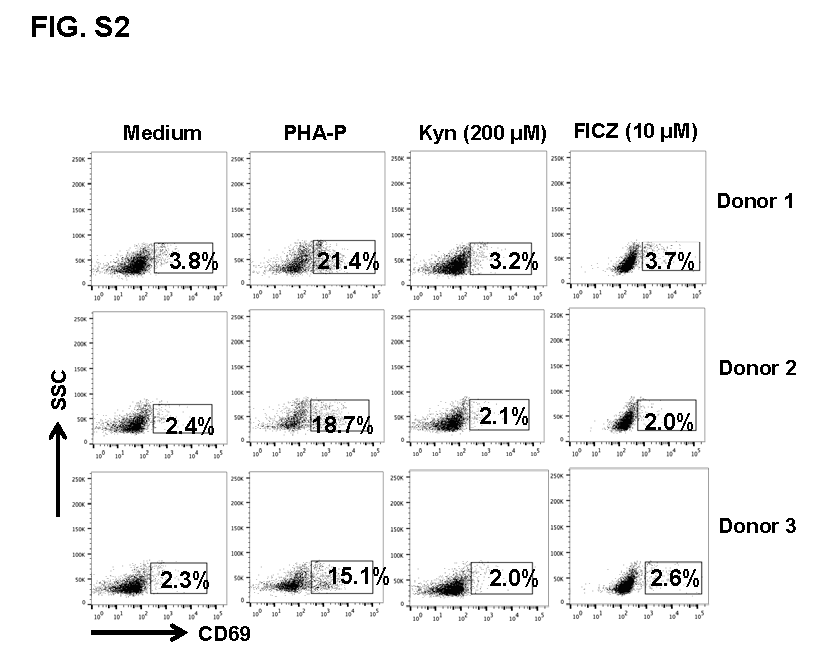

Supplement: FIG S2 [file mBio.02591-19-sf002.tif]

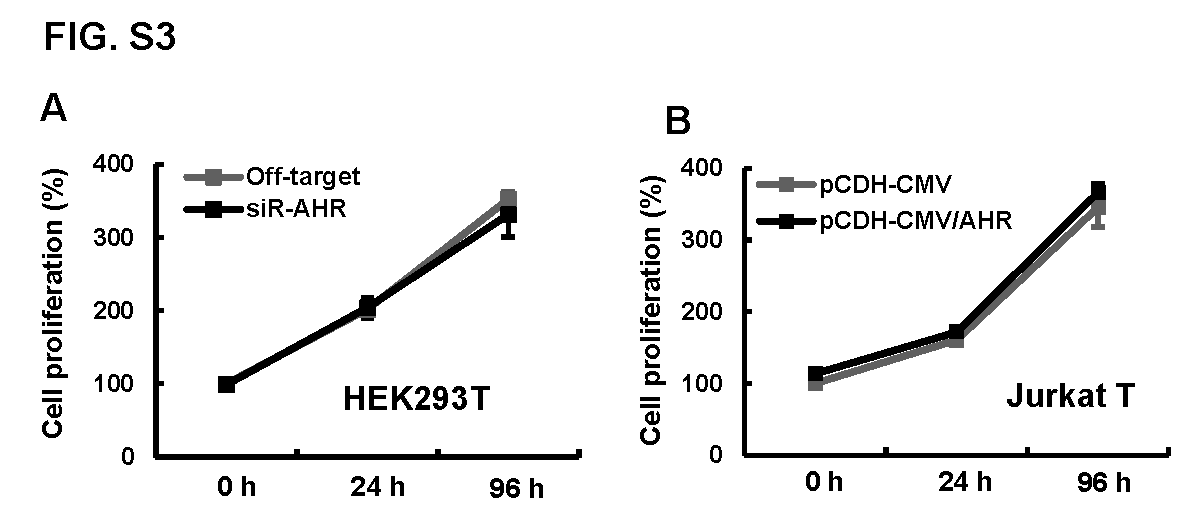

Supplement: FIG S3 [file mBio.02591-19-sf003.tif]
